# Prognostic Role of Pretreatment De Ritis Ratio (Aspartate Transaminase/Alanine Transaminase Ratio) in Urological Cancers: A Systematic Review and Meta-Analysis

**DOI:** 10.3389/fonc.2020.01650

**Published:** 2020-09-04

**Authors:** Shiqiang Su, Lizhe Liu, Chao Li, Jin Zhang, Shen Li

**Affiliations:** ^1^Department of Urology, Shijiazhuang People's Hospital, The No.1 Hospital of Shijiazhuang, Shijiazhuang, China; ^2^Department of Pathophysiology, Hebei Medical University, Shijiazhuang, China

**Keywords:** urological cancer, aspartate transaminase, alanine transaminase, prognosis, meta-analysis

## Abstract

**Background:** To examine the potential prognostic significance of pretreatment De Ritis ratio (aspartate transaminase/alanine transaminase ratio) in urological cancers, including upper tract urothelial cancer (UTUC), renal cell carcinoma (RCC), prostate cancer (PCa), bladder cancer (BCa).

**Methods:** Potential literatures were searched with PubMed, Embase, Cochrane Library, and Web of Science in December 2019. Merged hazard ratios (HRs) and 95% confidence intervals (CIs) were used to evaluate the associations.

**Results:** Totally, 15 studies with 8,565 patients were included. Merged results showed that an elevated pretreatment De Ritis ratio was correlated with poorer OS (HR 1.80, 95% CI 1.61–2.01), CSS (HR 2.15, 95% CI 1.80–2.56), PFS (HR 1.57, 95% CI 1.34–1.85), BRFS (HR 1.67, 95% CI 1.11–2.53) for urological cancers. Subgroup analyses by cancer type for OS found that De Ritis ratio can be a predictor in UTUC (HR 1.91, 95% CI 1.57–2.33), RCC (HR 1.74, 95% CI 1.47–2.07), and BCa (HR 1.80, 95% CI 1.43–2.27). Similar results could be found for CSS (UTUC: HR 2.46, 95% CI 1.93–3.13; RCC: HR 1.90, 95% CI 1.46-2.47; BCa: HR 2.71, 95% CI 1.39-5.31) and PFS (UTUC: HR 1.59, 95% CI 1.15–2.20; RCC: HR 1.52, 95% CI 1.26–1.83; BCa: HR 1.79, 95% CI 1.18–2.72). There was no publication bias among these included studies.

**Conclusions:** Pretreatment De Ritis ratio was a significant predictor for OS, CSS, PFS and BRFS in urological cancers, indicating that it could be a promising prognostic factor during clinical practice.

## Introduction

Urological cancers, mostly consisting of upper tract urothelial cancer (UTUC), renal cell carcinoma (RCC), prostate cancer (PCa), and bladder cancer (BCa), stand for a large proportion of adult malignances, especially for male patients. These four kinds of tumor belong to the top ten most commonly diagnosed malignances in the United States, 2019 (1). In general, most urological cancers were identified in localized stage. Surgical resection is the primary treatment option, and can obtain satisfactory results. However, there were still a part of patients experiencing metastatic disease at the time of initial diagnosis, moreover, most localized tumors will finally turn into recurrent or metastatic disease. Over the past decade, targeted therapy remained the mainstream treatment for metastatic urological cancers. Recently, the advent of sipuleucel-T based immunotherapy (2) and new molecular target agents (3, 4) further improved the prognosis of patients with metastatic disease. Nevertheless, the long-time survival is still unsatisfactory for these tumors. Therefore, it is important to explore prognostic factors in these patients, which may guide the treatment of urological cancers.

Aspartate aminotransferase (AST) and alanine aminotransferase (ALT) are long-familiar metabolic enzymes which can be generated by cancer and non-cancer cells. Previous studies have found the important prognostic role of these two enzymes in many kinds of malignances, including colorectal cancer, lung cancer, breast cancer, pancreatic cancer, and so on (5). The De Ritis ratio (AST/ALT) was firstly introduced by De Ritis et al. (6) to distinguish etiology of acute hepatitis about 50 years ago, and then became effective biomarker for kinds of hepatic diseases (7). Moreover, increasing evidence identified that De Ritis ratio can be an independent prognosis predictor for many kinds of malignances, such as hepatocellular carcinoma (8), head and neck cancer (9). More recently, the prognostic role of De Ritis ratio in genitourinary malignances has gained a lot of interest, and De Ritis ratio has been identified to be correlated with oncological outcomes for urological cancers. However, inconsistent results were reported. Hence, we aimed to systematical review the published literatures, and investigate De Ritis ratio as prognosis predictor in urological cancers, so as to provide high-level evidence for this issue.

## Materials and Methods

### Literature Search

In order to examine the prognostic significance of De Ritis ratio in urological cancers, the related literatures were searched with PubMed, Embase, Cochrane Library and Web of Science in December 2019. Strategy of searching was constituted the following major terms: “De Ritis ratio” (e.g., “De Ritis ratio,” “AST/ALT ratio,” “aspartate aminotransferase to alanine aminotransferase ratio,” “aspartate transaminase/alanine transaminase ratio”), “urological cancer” (e.g., “urological cancer,” “urothelial cancer,” or “transitional cell cancer”, “renal cancer,” “prostate cancer,” “bladder cancer”), and “survival” (e.g., “survival,” “prognosis,” “outcome,” “progression,” “recurrence,” “mortality”). A manual screen of literature references was also performed for related studies. During the process of literature searching, no language restriction was set. The current study was performed according to the guidelines of PRISMA criteria (10), and the protocol was registered (CRD 42019142310).

### Inclusion/Exclusion Criteria

All included studied focused on the prognostic significance of De Ritis ratio in genitourinary malignances. They also satisfied the next conditions: (1) patients were histopathologically diagnosed with urological cancers, including upper tract urothelial cancer (UTUC), renal cell carcinoma (RCC), prostate cancer (PCa), bladder cancer (BCa); (2) presented the correlation of pretreatment De Ritis ratio with specific endpoints, including overall survival (OS), cancer-specific survival (CSS), progression-free survival (PFS) for all urological cancers, and biochemical recurrence-free survival (BRFS) for PCa; (3) directly provided hazard ratios (HRs) and their 95% confidence interval (CI); (4) delivered in peer-review journals.

Exclusion criteria were as follows: (1) didn't provide critical data, such as HRs and 95% CIs for study endpoints; (2) studies about basic research; (3) letters, abstracts, opinions, reviews, and case report; (4) didn't analyze De Ritis ratio as dichotomous variable; (5) duplicated studies based on the same cohort, and in this case, the most comprehensive and latest study was chosen.

### Data Extraction and Quality Evaluation

The literature searching was independently conducted by two researchers. The disparities were resolved by a senior investigator. The detailed data was obtained from included literatures: the first authors' name, publication year, region of patients, study design, sample size, mean or median age of patients, cancer type and stage, details of management, value of cut-off, ways of determining the cut-off value, source of HRs and 95% CIs, oncological endpoints. Moreover, HRs and their 95% CIs for each endpoint were obtained from included literatures. Quality evaluation of included literatures was performed with the Newcastle-Ottawa scale.

### Statistical Analysis

Stata 12.0 (STATA Corporation, College Station) was used for all statistical analyses. Heterogeneity among studies was checked with the Higgins *I*^2^ statistic and Cochran's Q test. If significant heterogeneity (*P* < 0.10 and *I*^2^ > 50%) existed, the pooled HRs and 95% CIs was calculated with a random-effect model. Otherwise, we adopted the fixed-effect model. Subgroup analyses were undertaken to explore the associations of De Ritis ratio with classifying variables for OS and CSS. If ten or more studies were included in a meta-analysis, visually funnel plots and quantitative Begg's and Egger's tests were both applied to test publication bias. Sensitivity analysis was performed by excluding each literature by turn to evaluate the reliability and stability of the outcomes.

## Results

### Searching Process and Characteristics of Studies

**Figure 1:**
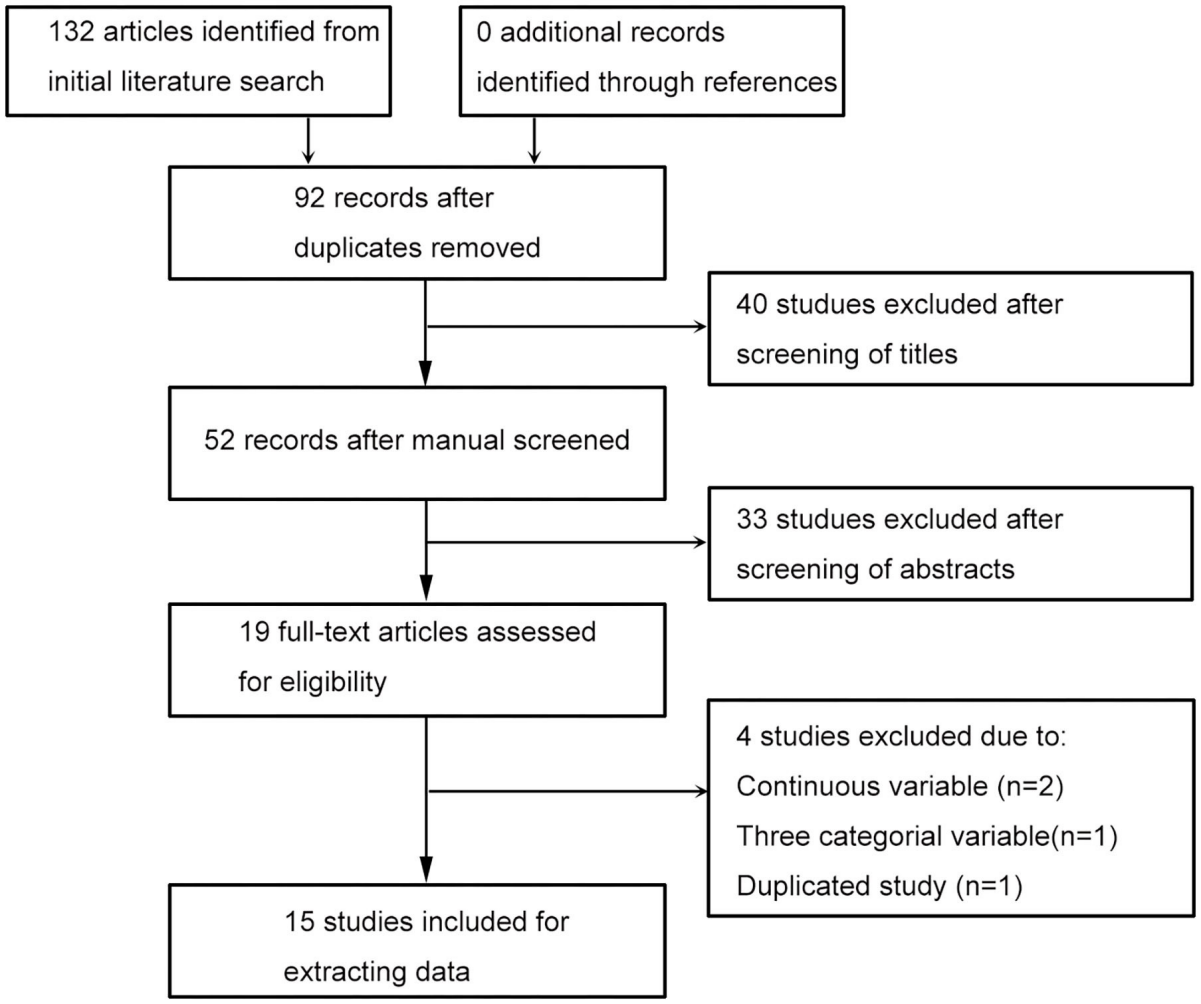
The flow diagram of literature selection.

The process of literature searching was presented in Figure 1. Database searching has obtained 132 articles, and no additional record has been identified through screening references of related literature. After removing 40 duplicates, 40 studies, and 33 studies were, respectively, excluded by literature titles and abstracts screening. Nineteen studies were evaluated for eligibility with full-text, three of them were got rid of because of continuous or three categorial variable, one of them was excluded due to duplicated study. Lastly, 15 studies (5, 11–24) were included for extracting data.

All literatures were recently published (2015–2019) with a retrospective design. Most objects of studies were Asian population. Totally 8,565 patients were analyzed in these studies, and the median size of patient number was 298 (IQR 135–698). The median and mean age of participates were, respectively, reported by eight and four studies. The median age ranged from 55 to 70 years, and the mean age ranged from 58.6 to 67 years. Of all studies, four focused on UTUC, six focused on RCC, two focused on PCa, three focused on BCa. Most patients were of non-metastatic disease, and treated by surgery (Table 1).

**Table 1:** Features of included studies.

**Study**	**Year**	**Country**	**Study**	**Case**	**Age (Years)**	**Cancer**	**Stage**	**Treatment**	**Cut-off**	**Determine the**	**COX**	**Survival Analysis**	**SQ**
			**Design**	**Number**		**Type**			**(g/L)**	**cut-off value**			
Yuk	2019	South Korea	RTP, SC	610	-	BCa	Non-metastatic	Surgery	1.1	ROC	mul	OS, CSS, PFS	8
Li	2019	China	RTP, SC	885	67.0 ± 10.6	UTUC	Non-metastatic	Surgery	1.23	ROC	mul	OS, CSS, PFS	8
Ikeda	2019	Japan	RTP, SC	243	61 (55–67)	RCC	Non-metastatic	Surgery	1.42	Youden index	mul	CSS	8
Ha	2019	South Korea	RTP, SC	118	69 (60–74)	BCa	Non-metastatic	Surgery	1.3	ROC	mul	OS, CSS, PFS	7
Nishikawa	2018	Japan	RTP, SC	135	69 (52–86)	UTUC	Non-metastatic	Surgery	1.3	Reported	mul	PFS	7
Miyake	2018	Japan	RTP, SC	74	-	PCa	Metastatic	Chemotherapy	1.35	Median	mul/uni	OS, BRFS	6
Kang	2018	South Korea	RTP, SC	158	58.6 ± 10.6	RCC	Metastatic	Targeted therapy	1.2	ROC	mul	OS, CSS	7
Canat	2018	Turkey	RTP, SC	298	61.5 ± 13.2	RCC	Non-metastatic	Surgery	1.5	ROC	uni	CSS	6
Wang	2017	China	RTP, SC	438	70 (65–74)	PCa	Non-metastatic	Surgery	1.325	ROC	uni/mul	OS, BRFS	7
Lee	2017	South Korea	RTP, MI	2965	55 (47–65)	RCC	Non-metastatic	Surgery	1.5	ROC	mul	OS, CSS, PFS	7
Lee	2017	South Korea	RTP, SC	623	65 (56–72)	UTUC	Non-metastatic	Surgery	1.5	ROC	mul	OS, CSS, PFS	7
Ishihara	2017	Japan	RTP, SC	118	-	RCC	Metastatic	Surgery	1.24	ROC	mul	OS, CSS	6
Gorgel	2017	Turkey	RTP, SC	153	61.7 ± 9.1	BCa	Non-metastatic	Surgery	1.3	ROC	mul	OS, CSS	6
Cho	2017	South Korea	RTP, MI	1049	69 (61–74)	UTUC	Non-metastatic	Surgery	1.6	ROC	mul/uni	OS, CSS, PFS	7
Bezan	2015	Austria	RTP, SC	698	65 (56–73)	RCC	Non-metastatic	Surgery	1.26	ROC	mul	OS, PFS	8

*SQ, study quality according to the Newcastle-Ottawa scale; RTP, retrospective; SC, single center; MI, multi-institutional; UTUC, upper tract urothelial cancer; BCa, bladder cancer; PCa, prostate cancer; RCC, renal cell carcinoma; ROC, receiver-operating characteristic; OS, overall survival; CSS, cancer-specific cancer; PFS, progression-free survival; BRFS, biochemical recurrence-free survival*.

### De Ritis Ratio and OS in Urological Cancers

**Figure 2:**
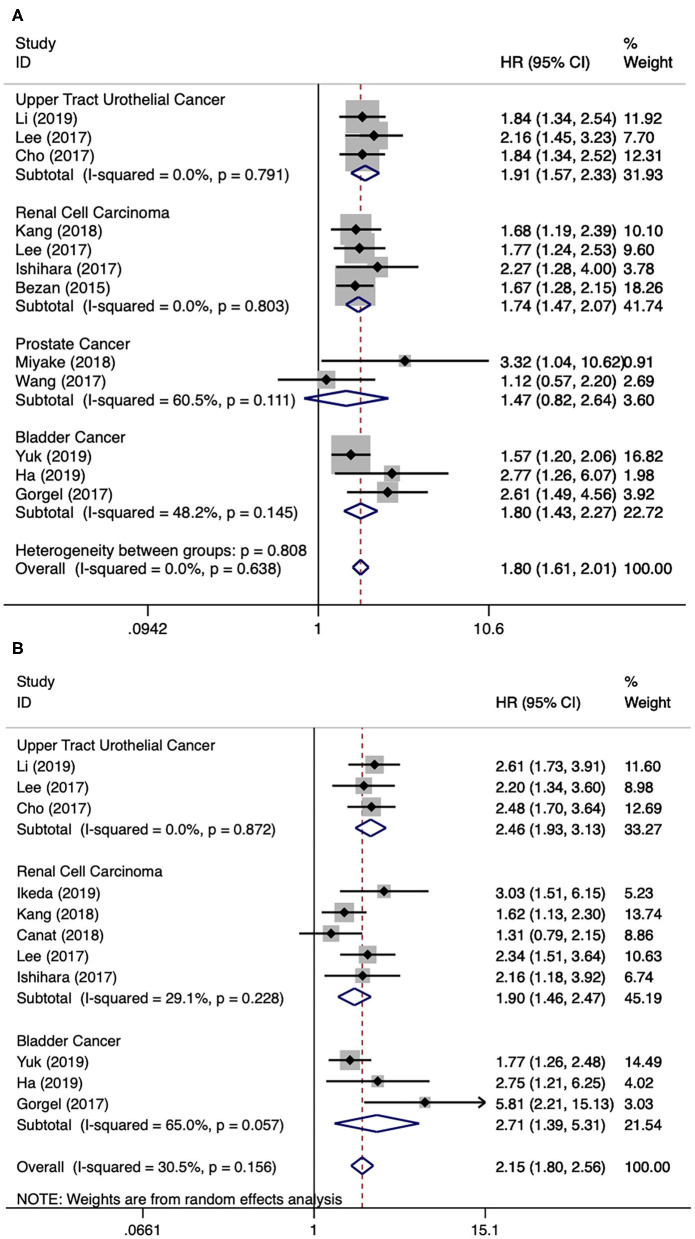
Forest plot reflects the association between De Ritis ratio and OS/CSS for urological cancers. **(A)** De Ritis ratio and OS; **(B)** De Ritis ratio and CSS.

There were twelve literatures for OS in urological cancers (5, 11, 12, 14, 15, 17–23). Since no obvious heterogeneity among these literatures (*I*^2^ = 0.0%, *p* = 0.638), a fixed model analysis was performed. The merged results presented that a higher level of AST/ALT ratio was correlated with poor OS for urological cancers (HR 1.80, 95% CI 1.61–2.01, *p* < 0.001). The subgroup analyses by cancer type have identified that a higher level of AST/ALT ratio was correlated with inferior OS for UTUC (HR 1.91, 95% CI 1.57–2.33, *p* < 0.001), RCC (HR 1.74, 95% CI 1.47–2.07, *p* < 0.001) and BCa (HR 1.80, 95% CI 1.43–2.27, *p* < 0.001), but not for PCa (HR 1.47, 95% CI 0.82–2.64, *p* = 0.195) (Figure 2A). Besides cancer type, subgroup analyses for OS were also performed by many other variables, including publication year, continent, country, sample size, and so on. The results remained significant by these subgroup variables (Table 2).

**Table 2:** Subgroup analysis of overall survival and cancer-specific survival.

**Subgroup**	**Studies**	**HR (95% CI)**	***P*-value**	**Heterogeneity**	
				***I^**2**^* (%)**	***P*-value**
**OVERALL SURVIVAL**					
Year of publication					
2018–2019	5	1.74 (1.47–2.07)	<0.001	0.0	0.515
2015–2017	7	1.84 (1.59–2.12)	<0.001	0.0	0.498
**Continent**					
Asia	10	1.79 (1.58–2.04)	<0.001	0.0	0.660
Europe	2	1.94 (1.28–2.95)	0.002	51.1	0.153
**Country**					
China	2	1.68 (1.26–2.24)	<0.001	41.8	0.190
South Korea	6	1.78 (1.54–2.06)	<0.001	0.0	0.683
Japan	2	2.44 (1.47–4.08)	0.001	0.0	0.564
**Site of carcinoma**					
Upper Tract Urothelial Cancer	3	1.91 (1.57–2.33)	<0.001	0.0	0.791
Renal Cell Carcinoma	4	1.74 (1.47–2.07)	<0.001	0.0	0.803
Prostate Cancer	2	1.47 (0.82–2.64)	0.195	60.5	0.111
Bladder Cancer	3	1.80 (1.43–2.27)	<0.001	48.2	0.145
**Sample size**					
>200	7	1.73 (1.53–1.96)	<0.001	0.0	0.718
<200	5	2.09 (1.64–2.66)	<0.001	0.0	0.511
Cancer stage					
Non–metastatic	9	1.78 (1.58–2.01)	<0.001	0.0	0.543
Metastatic	3	1.89 (1.42–2.53)	<0.001	0.0	0.420
Cut–off value					
>1.3	5	1.84 (1.52–2.23)	<0.001	0.0	0.439
≤1.3	7	1.77 (1.55–2.03)	<0.001	0.0	0.549
NOS score					
≥7	9	1.74 (1.55–1.95)	<0.001	0.0	0.748
<7	3	2.52 (1.73–3.67)	<0.001	0.0	0.835
**CANCER–SPECIFIC SURVIVAL**					
Year of publication					
2018–2019	6	1.90 (1.58–2.28)	<0.001	36.6	0.162
2017	5	2.45 (1.96–3.07)	<0.001	0.0	0.478
**Continent**					
Asia	9	2.14 (1.84–2.48)	<0.001	0.0	0.581
Europe	2	2.60 (0.61–11.15)	0.198	86.2	0.007
**Country**					
South Korea	6	2.02 (1.71–2.40)	<0.001	0.0	0.503
Japan	2	2.49 (1.58–3.93)	<0.001	0.0	0.471
Turkey	2	2.60 (0.61–11.15)	0.198	86.2	0.007
**Site of carcinoma**					
Upper Tract Urothelial Cancer	3	2.46 (1.93–3.13)	<0.001	0.0	0.872
Renal Cell Carcinoma	5	1.87 (1.51–2.31)	<0.001	29.1	0.228
Bladder Cancer	3	2.71 (1.39–5.31)	0.004	65.0	0.057
**Sample size**					
> 200	7	2.13 (1.81–2.52)	<0.001	20.3	0.274
<200	4	2.39 (1.48–3.85)	<0.001	55.6	0.080
Cancer stage					
Non–metastatic	9	2.21 (1.89–2.60)	<0.001	32.6	0.157
Metastatic	2	1.74 (1.28–2.37)	<0.001	0.0	0.415
Cut–off value					
>1.3	5	2.18 (1.76–2.69)	<0.001	25.7	0.250
≤1.3	6	2.05 (1.70–2.48)	<0.001	43.4	0.116
**NOS score**					
≥7	8	2.14 (1.83–2.50)	<0.001	0.0	0.472
<7	3	2.31 (1.10–4.83)	0.027	73.3	0.024

*HR, hazard ratio; CI, confidence interval; NOS, Newcastle-Ottawa Scale*.

### De Ritis Ratio and CSS in Urological Cancers

There were 11 literatures for CSS in urological cancers (5, 11, 12, 15, 16, 18–21, 23, 24). The merged results presented that a higher level of AST/ALT ratio was correlated with poor CSS for urological cancers (HR 2.15, 95% CI 1.80–2.56, *p* < 0.001). The subgroup analyses by cancer type have identified that a higher level of AST/ALT ratio was correlated with inferior CSS for UTUC (HR 2.46, 95% CI 1.93–3.13, *p* < 0.001), RCC (HR 1.90, 95% CI 1.46–2.47, *p* < 0.001) and BCa (HR 2.71, 95% CI 1.39–5.31, *p* < 0.001) (Figure 2B). Besides cancer type, subgroup analyses for CSS were also performed by many other variables, including publication year, continent, country, sample size, and so on. Except for patients from Turkey, the results remained significant by these subgroup variables (Table 2).

### De Ritis Ratio and PFS/BRFS in Urological Cancers

**Figure 3:**
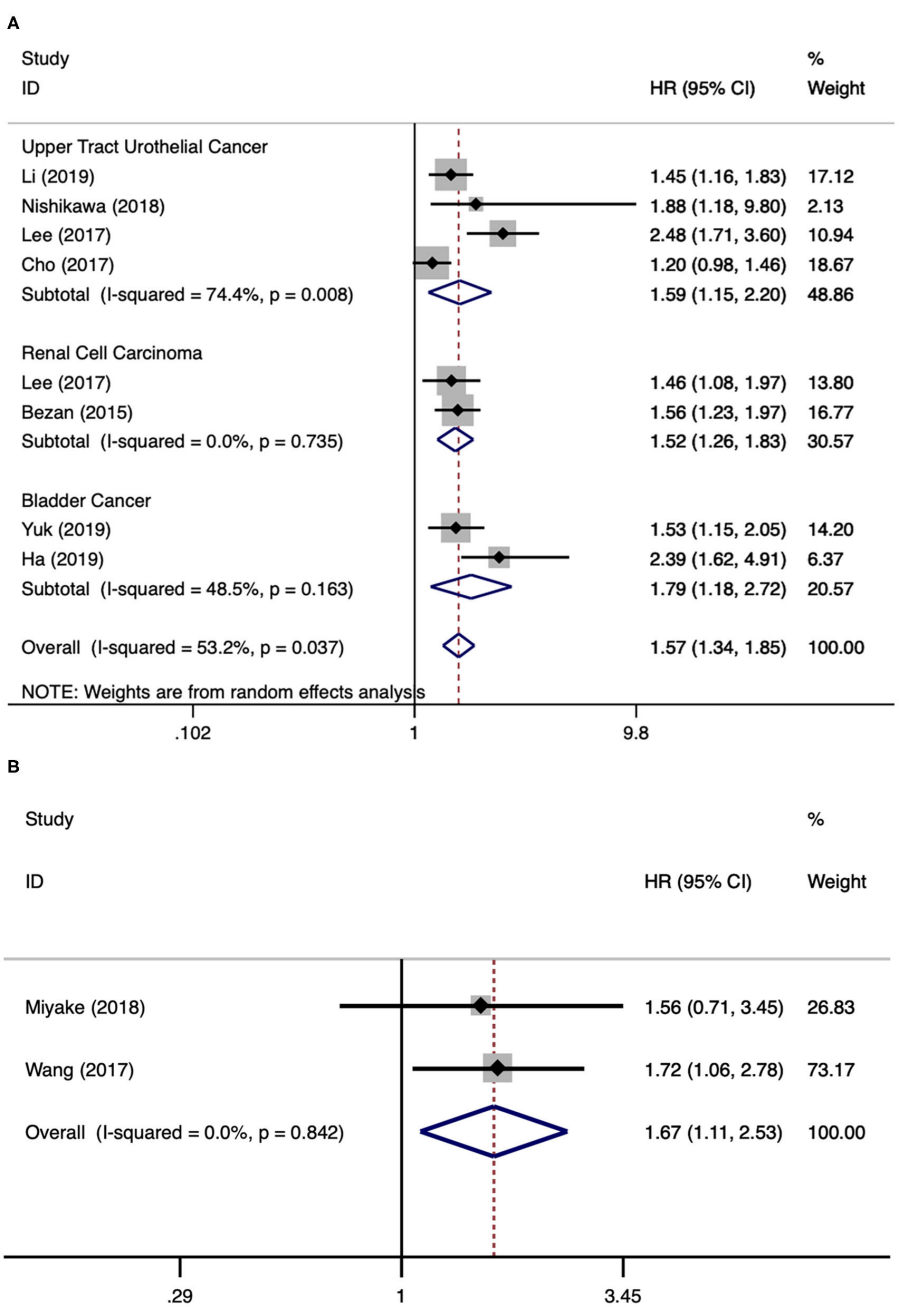
Forest plot reflects the association between De Ritis ratio and PFS/BRFS for urological cancers. **(A)** De Ritis ratio and PFS; **(B)** De Ritis ratio and BRFS.

There were eight literatures for PFS in urological cancers (5, 11–13, 18, 21–23). Since significant heterogeneity existed among these studies (*I*^2^ = 53.2%, *p* = 0.037), a random model analysis was performed. The merged results presented that a higher level of AST/ALT ratio was associated with poor PFS for urological cancers (HR 1.57, 95% CI 1.34–1.85, *p* < 0.001). The subgroup analyses by cancer type have found that a higher level of AST/ALT ratio was associated with poor PFS for UTUC (HR 1.59, 95% CI 1.15–2.20, *p* = 0.005), RCC (HR 1.52, 95% CI 1.26–1.83, *p* < 0.001), and BCa (HR 1.79, 95% CI 1.18–2.72, *p* < 0.001) (Figure 3A). There were two literatures for BRFS in PCa (14, 17). Since no obvious heterogeneity was identified among these studies (*I*^2^ = 0.0%, *p* = 0.842), a fixed model analysis was performed. The merged results presented that a higher level of De Ritis ratio was correlated with poor BRFS for PCa (HR 1.67, 95% CI 1.11–2.53, *p* = 0.014) (Figure 3B).

### Publication Bias

**Figure 4:**
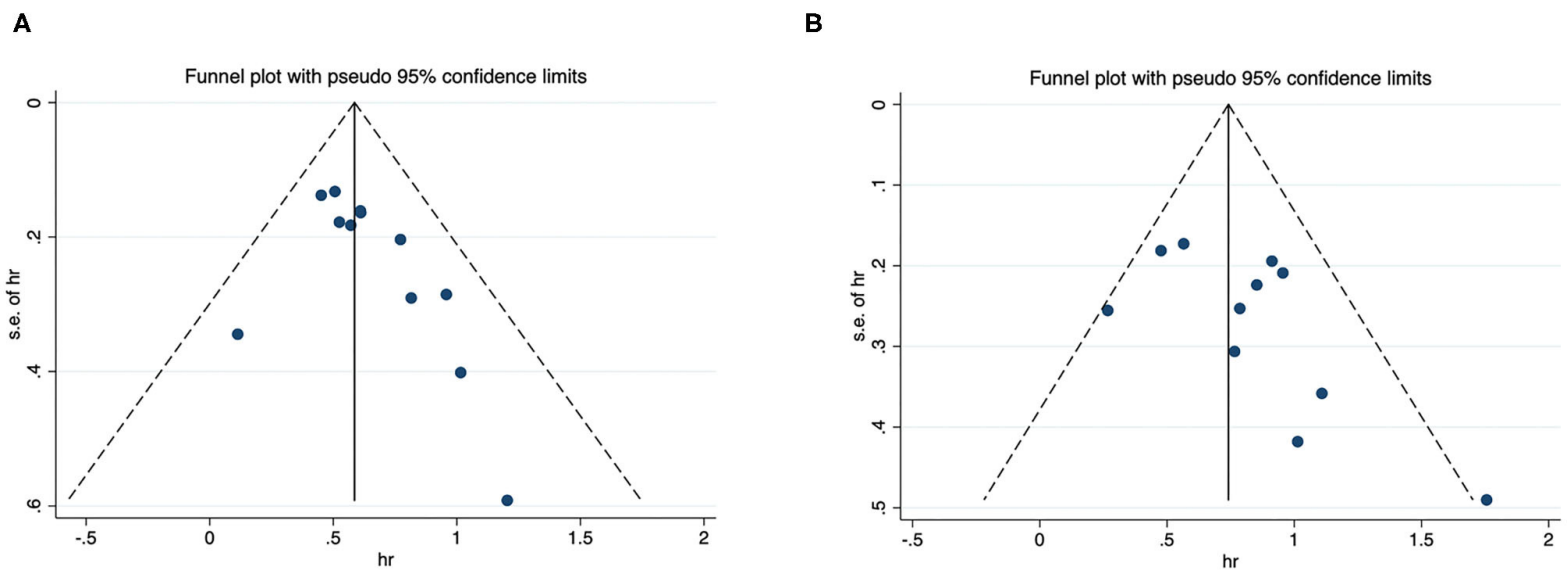
Funnel plot for publication bias. **(A)** correlation of De Ritis ratio with OS in urological cancers; **(B)** correlation of De Ritis ratio with CSS in urological cancers.

Funnel plots for OS and CSS were presented in Figure 4, and they were visually asymmetry. Quantitatively Begg's and Egger's test also showed low possibility of publication bias for OS (*P* = 0.119 and 0.116) and CSS (*P* = 0.376 and 0.208).

### Sensitivity Analysis

**Figure 5:**
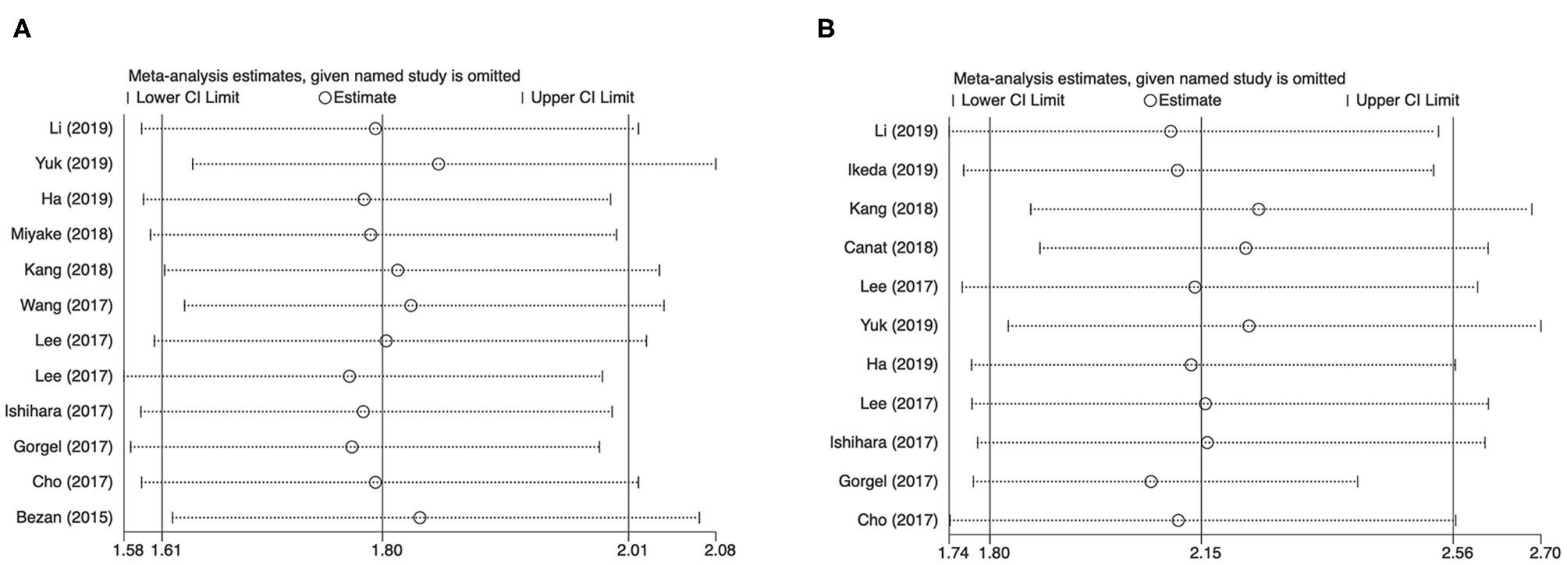
Results of sensitivity analysis. **(A)** correlation of De Ritis ratio with OS in urological cancers; **(B)** correlation of De Ritis ratio with CSS in urological cancers.

Each study was excluded each time to validate the stability of results. The merged HRs for De Ritis ratio and OS or CSS in cases with urological cancers were not obviously altered (Figure 5).

## Discussion

Genitourinary cancers represent a large proportion of adult malignances, especially for men. The most common types of urological cancers are UTUC, RCC, PCa, and BCa (1). With the improvement of surgical skills and the advent of molecular targeted therapy and immunotherapy, the oncological outcomes of genitourinary cancers had got a significant progress (2–4). However, postoperative recurrence and distant metastasis also are high-frequency events for urological cancers, and the prognosis for these patients was unfavorable. Therefore, it is needed to explore the prognosis biomarkers for urological cancers, which may guide the clinical treatments.

During clinical practice, AST and ALT were applied to assess liver function, and to recognize liver diseases such as viral and alcoholic hepatitis. In 1957, De Ritis et al. (6) initially applied the De Ritis ratio (AST/ALT) to distinguish the etiology of acute hepatitis. Meanwhile, a large number of studies in recent years have shown that AST/ALT has important prognostic value in various urinary system tumors, including UTUC, RCC, PCa, and BCa. This study tried to systematic review related published studies and applied the method of meta-analysis to evaluate the prognostic significance of De Ritis ratio in genitourinary malignances. Based on the high-quality studies, after merging relevant results, a higher pretreatment De Ritis ratio was identified to be significantly associated with poor OS, CSS, PFS and BRFS. The results for these four endpoints remained significant for UTUC, RCC, PCa, and BCa, respectively. The subgroup analyses in terms of different variables for overall survival and cancer-specific survival didn't change the direction of results. Publication bias checking and sensitivity analyses have also validated the reliability and robust of our results. AST and ALT are commonly applied hematological index in clinical practice, which are simply and easily measured with low cost. Hence, De Ritis ratio can be widely used as an effective prognostic marker in clinical diagnosis and treatment of urological cancers.

More recently, a meta-analysis has studied De Ritis ratio as prognosis predictor in solid cancers. They have found that a higher level of AST/ALT ratio was significantly correlated with poor OS, CSS, and RFS in all cancer types (25). However, there were several limitations for this study. Firstly, tumors from different systems may have different pathogenesis, it seems unreasonable to analyze them together. Secondly, most included studies analyzed De Ritis ratio as a dichotomous variable, however, Gu et al. (26) analyzed De Ritis ratio as a continuous variable. It seems unreasonable to analyze them together. Therefore, our study didn't include the study by Gu et al. (26). Moreover, our study was performed later, we can include new evidence. Hence, our study can provide the latest and most comprehensive evidence for the prognostic value of De Ritis ratio in urological cancers.

AST can be synthesized in kinds of tissues, while ALT is mainly synthesized in liver (27). Therefore, damage of tissue due to tumor cells or metabolic changes in rapidly proliferate tumor cells can raise the AST level in peripheral blood, but the ALT level does not change much. Further, literatures have reported that AST and ALT in tumor cells catalyze changes in the end products of cellular metabolic pathways (28). Therefore, AST/ALT ratio level have theoretical basis as potential prognostic factor in cancer patients. The specific mechanism of the relationship between De Ritis ratio and prognosis of urological cancers is still not clear, and further research is needed. One possible hypothesis is that aspartic and alanine transferase play important roles in the metabolism of urological cancers. Metabolic remodeling to ensure adequate energy and break the key blocks of tumor cell proliferation are important tumor characteristics (29). Compared with the oxidative phosphorylation of mitochondria in normal cells, the aerobic glycolysis rate of tumor cells was significantly increased after mitochondrial dysfunction (30). Besides “Warburg effect” this particular phenomenon, tumor cells exhibit a significant higher synthetic rate of nucleotide and amino acid to meet the needs of highly proliferative state (31). An important TCGA study found that metabolic changes exert a vital role in tumor progression and survival outcomes of RCC (32). Meanwhile, a recent study found that RCC was characterized by extensive recombination of cell metabolism, and these metabolic metastases were closely related to tumor progression and metastasis (33). In summary, these results highlighted the relationship between changes in cell metabolism and the biology of urological cancers. Furthermore, ALT relocates NADH into mitochondria through malic acid-aspartic acid shuttle pathway, which plays an important role in glycolysis. Therefore, aspartic and alanine transferases are highly likely to be involved in glycolytic mechanisms of urological cancers, although our apprehension of the exact mechanisms is constricted.

Of course, our study has some limitations. First, almost all the studies were single-center retrospective designed, and part of hazard ratios and corresponding 95% confidence intervals extracted from univariate analysis results, and some uncontrollable bias may exist. Second, because this study only included published literature, there may be publication bias. Third, we tried to obtain comprehensive data. However, there is still little literature on some outcomes, especially biochemical recurrence-free survival. Finally, no included studies analyzed patients from America or Africa, which may cause publication bias, and it also can be a weakness limiting generalizability of the findings.

## Conclusions

The results found that a higher pretreatment De Ritis ratio was significantly correlated with lower OS, CSS, PFS and BRFS in urological cancers. De Ritis ratio level before treatment can be applied as a significant prognostic factor to guide clinical treatment of urological cancers.

## Data Availability Statement

All datasets presented in this study are included in the article.

## Author Contributions

SS and CL: conception and design and manuscript writing/editing. SS, LL, and JZ: data collection or management. SS and SL: data analysis. All authors read and approved the final manuscript. All authors contributed to the article and approved the submitted version.

## Conflict of Interest

The authors declare that the research was conducted in the absence of any commercial or financial relationships that could be construed as a potential conflict of interest.
